# The Detrimental Action of Adenosine on Glutamate-Induced Cytotoxicity in PC12 Cells Can Be Shifted towards a Neuroprotective Role through A_1_AR Positive Allosteric Modulation

**DOI:** 10.3390/cells9051242

**Published:** 2020-05-18

**Authors:** Fabrizio Vincenzi, Silvia Pasquini, Stefania Gessi, Stefania Merighi, Romeo Romagnoli, Pier Andrea Borea, Katia Varani

**Affiliations:** 1 Department of Morphology, Surgery and Experimental Medicine, Pharmacology Section, University of Ferrara, 44121 Ferrara, Italy; psqslv@unife.it (S.P.); gss@unife.it (S.G.); mhs@unife.it (S.M.); vrk@unife.it (K.V.); 2 Department of Chemical and Pharmaceutical Sciences, University of Ferrara, 44121 Ferrara, Italy; rmr@unife.it; 3 University of Ferrara, 44121 Ferrara, Italy; bpa@unife.it

**Keywords:** adenosine, glutamate cytotoxicity, positive allosteric modulation, neuroprotection, apoptosis

## Abstract

Glutamate cytotoxicity is implicated in neuronal death in different neurological disorders including stroke, traumatic brain injury, and neurodegenerative diseases. Adenosine is a nucleoside that plays an important role in modulating neuronal activity and its receptors have been identified as promising therapeutic targets for glutamate cytotoxicity. The purpose of this study is to elucidate the role of adenosine and its receptors on glutamate-induced injury in PC12 cells and to verify the protective effect of the novel A_1_ adenosine receptor positive allosteric modulator, TRR469. Flow cytometry experiments to detect apoptosis revealed that adenosine has a dual role in glutamate cytotoxicity, with A_2A_ and A_2B_ adenosine receptor (AR) activation exacerbating and A_1_ AR activation improving glutamate-induced cell injury. The overall effect of endogenous adenosine in PC12 cells resulted in a facilitating action on glutamate cytotoxicity, as demonstrated by the use of adenosine deaminase and selective antagonists. However, enhancing the action of endogenous adenosine on A_1_ARs by TRR469 completely abrogated glutamate-mediated cell death, caspase 3/7 activation, ROS production, and mitochondrial membrane potential loss. Our results indicate a novel potential therapeutic strategy against glutamate cytotoxicity based on the positive allosteric modulation of A_1_ARs.

## 1. Introduction

Glutamate cytotoxicity is one of the main contributing factors in different pathologies of the central nervous system (CNS), including neurodegenerative diseases, cerebral ischemia, and traumatic brain injury. Excitotoxicity results from excessive release of the neurotransmitter glutamate, which in turn stimulates different subtypes of synaptic and extra-synaptic receptors in neurons, leading to Ca^2+^ overload, oxidative stress, mitochondrial dysfunctions and eventually neuronal death [1]. Given the relevant role of glutamate receptors in cytotoxicity, the initial therapeutic approach was based on the use of glutamate receptors antagonists, in particular *N*-Methyl-d-Aspartate (NMDA) receptor blockers [2]. However, glutamate receptors antagonists failed to show efficacy in clinical trials of stroke or traumatic brain injury. In addition, glutamate receptor blockade seems to interfere with physiological neuronal function and cause relevant adverse effects at potentially therapeutic concentrations [3,4]. Glutamate toxicity can also occur through a Ca^2+^- and receptor-independent, oxidative-mediated cell injury. In particular, it has been postulated that glutamate accumulation can reverse the action of the cystine (CySS)/glutamate antiporter (Xc-), inhibiting the uptake of CySS [5]. As CySS is intracellularly reduced to cysteine for glutathione (GSH) synthesis, by depleting neurons of CySS and eventually GSH, elevated extracellular concentrations of glutamate leads to free radical accumulation and cell death by oxidative stress [6]. Excitotoxicity and glutamate oxidative toxicity are causal factors in the neuropathology of several neurological disorders. Therefore, new therapeutic strategies aimed at reducing glutamate-induced cytotoxicity are strongly warranted.

Adenosine is a ubiquitous endogenous purine nucleoside that regulates almost all aspects of cellular and tissue physiology through the interaction with four G protein-coupled receptors (GPCR) named A_1_, A_2A_, A_2B_, and A_3_ adenosine receptors (ARs) [7]. In the CNS, by activating A_1_ARs, adenosine reduces Ca^2+^ influx and increases the conductance of K^+^ and Cl^−^ ions thereby inducing presynaptic inhibition and the reduction of neuronal excitability and excitatory neurotransmitters release [8,9]. Several papers in the literature report the neuroprotective effect of A_1_AR activation on glutamate-induced cytotoxicity and related disorders such as cerebral ischemia [10,11,12,13]. On the other hand, activation of A_2A_ARs has been shown to exert a detrimental effect on various neurodegenerative diseases mainly through increasing glutamate release, suggesting that their blockade could represent a valuable tool for excitotoxicity-related pathologies [14,15,16]. Among ARs, the A_2B_ subtype is the least studied and remains the most enigmatic even if recent studies indicated that selective antagonists may exert neuroprotective effects in brain ischemia [17]. The role of A_3_ARs in neuroprotection is controversial although its activation has been reported to exert neuroprotective effect against glutamate cytotoxicity in retinal ganglion cells [18,19].

Despite the neuroprotective potential of A_1_AR activation, the use of A_1_AR full agonist in the clinic has been dampened by cardiovascular side effects and receptor desensitization [20]. A possible approach to circumvent A_1_AR agonist-mediated adverse effects and receptor desensitization is the use of positive allosteric modulators (PAMs). The possibility offered by PAMs to target the receptor at the allosteric site might increase tissue specificity because the drug would act in concert with locally increased adenosine under pathological conditions and have little effect on sites where there are low basal adenosine levels [21]. To exploit their advantages, we have designed, synthesized, and characterized different series of A_1_AR PAMs, some of which represent the most potent and effective so far synthesized [22,23,24,25,26,27]. Among these, we have demonstrated the antinociceptive and anxiolytic-like properties of the novel A_1_AR positive allosteric modulator (2-Amino-4-[(4-(phenyl)piperazin-1-yl)methyl]-5-(4-fluorophenyl)thiophen-3-yl)-(4-chlorophenyl)methanone (TRR469) in different in vivo models [28,29,30].

The present study aimed to determine the role of adenosine and its receptor on glutamate-induced cytotoxicity in PC12 cells and to verify the cytoprotective effect of the positive allosteric modulator TRR469 by potentiating the action of endogenous adenosine on A_1_ARs.

## 2. Materials and Methods

### 2.1. Chemicals and Reagents

The following ARs agonists and antagonists were purchased from Tocris (Bristol, UK): NECA (5′-(*N*-Ethyl-carboxamido)adenosine); CGS15943 (9-Chloro-2-(2-furanyl)-[1,2,4]triazolo[1,5-c]quinazolin-5-amine); DPCPX (1,3-Dipropyl-8-cyclopentylxanthine); SCH442416 (2-(2-Furanyl)-7-[3-(4-methoxy-phenyl)propyl]-7*H*-pyrazolo[4,3-e][1,2,4]triazolo[1,5-c]pyrimidin-5-amine); PSB603 (8-(4-(4-(4-Chlorophenyl)piperazide-1-sulfonyl) phenyl)-1-propylxanthine). CCPA (2-Chloro-N6-cyclopentyladenosine) and MRS1523 (3-Propyl-6-ethyl-5-[(ethylthio)carbonyl]-2 phenyl-4-propyl-3-pyridine carboxylate), adenosine deaminase (ADA), l-glutamic acid monosodium salt monohydrate, forskolin, KT5720, tert-Butyl hydroperoxide (TBHP), *N*-acetylcysteine (NAC) were obtained from Sigma-Aldrich (St. Luis, MO, USA). Annexin V AlexaFluor^TM^ 488 Ready Flow Conjugate, SYTOX™ AADvanced™ Dead Cell Stain, CellROX™ Green Reagent, MitoTracker™ Red CMXRos were purchased from Life Technologies (Monza, Italy). The A_1_AR positive allosteric modulator TRR469 was previously synthesized and characterized (compound 4ad in [23]).

### 2.2. Cell Culture

Rat pheochromocytoma PC12 cells were purchased from American Type Culture Collection (Manassas, VA, USA) and were maintained in DMEM High Glucose medium supplemented with 5% FBS, 10% horse serum, 2 mM l-glutamine, 100 U/mL penicillin and 100 μg/mL streptomycin in a humidified atmosphere (5% CO_2_) at 37 °C [31]. Undifferentiated PC12 cells were used as one of the most common cell line models to study glutamate cytotoxicity [32]. For glutamate exposure, a freshly prepared 1 M l-glutamic acid monosodium salt monohydrate solution (pH 7.0) was used.

### 2.3. Apoptosis Evaluation

PC12 cells were seeded in 6 well plates at a density of 2 × 10^5^ cells/well and incubated overnight in a humidified atmosphere (5% CO_2_) at 37 °C. Cells were pre-treated with ADA for 30 min and with ARs agonist, antagonist, TRR469, and forskolin for 15 min before glutamate exposure for an additional 24 h. At the end of incubation, cells were detached using Accutase (Life Technologies, Monza, Italy) and subsequently stained with Annexin V Alexa Fluor™ 488 Ready Flow Conjugate to 1 × 10^5^ cells in 100 µL of Annexin Binding Buffer (Life Technologies). Cells were then incubated for 5 min at 25 °C, followed by the addition of 1 µM SYTOX™ AADvanced™ Dead Cell Stain. Data were acquired on an Attune NxT Flow Cytometer (Thermo-Fisher Scientific, Paisley, UK) equipped with a 488 nm laser for excitation. Fluorescence emission was collected using a 530/30 BP filter and a 695/40 BP filter for Annexin V Alexa Fluor™ 488 and SYTOX™ AADvanced™, respectively. PC 12 cells were gated according to physical parameters and cell aggregates were removed from the analysis.

### 2.4. Activation of Caspases 3/7

PC12 cells were seeded in 6 well plates at a density of 2 × 10^5^ cells/well and incubated overnight in a humidified atmosphere (5% CO_2_) at 37 °C. Cells were pre-treated with TRR469 for 15 min before glutamate exposure for an additional 24 h. At the end of incubation, cells were detached using Accutase and subsequently stained by adding 1 µM of CellEvent™ Caspase-3/7 Green Detection Reagent (Thermo-Fisher Scientific) to 1 × 10^5^ cells in 1 mL of PBS and incubated for 30 min at 37 °C. Data were acquired on an Attune NxT Flow Cytometer equipped with a 488 nm laser for excitation and fluorescence emission was collected using a 530/30 BP filter. PC 12 cells were gated according to physical parameters and cell aggregates were removed from the analysis.

### 2.5. ROS Production Evaluation

PC12 cells were seeded in 6 well plates at a density of 2 × 10^5^ cells/well and incubated overnight in a humidified atmosphere (5% CO_2_) at 37 °C. Cells were pre-treated with TRR469 for 15 min before glutamate exposure for an additional 6 h. For the positive control, cells were treated with 200 μM tert-Butyl hydroperoxide (TBHP) for 1 h at 37 °C. For the negative control, cells were pre-treated with 2 mM *N*-acetylcysteine (NAC) for 1 h at 37 °C, before the exposure with 200 μM TBHP for an additional hour at 37 °C. At the end of the treatment period, 1 μM CellROX™ Green Reagent was added to the cells followed by an incubation of 45 min at 37 °C. Immediately after cell detachment, data were acquired on an Attune NxT Flow Cytometer equipped with a 488 nm laser for excitation and fluorescence emission was collected using a 530/30 BP filter. PC 12 cells were gated according to physical parameters and cell aggregates were removed from the analysis.

### 2.6. Mitochondrial Membrane Potential Evaluation

PC12 cells were seeded in 96 well plates at a density of 1 × 10^4^ cells/well and incubated overnight in a humidified atmosphere (5% CO_2_) at 37 °C. Cells were pre-treated with TRR469 for 15 min before glutamate exposure for an additional 24 h. At the end of incubation, cells were stained with 200 nM MitoTracker™ Red CMXRos, a red-fluorescent dye the accumulation of which is dependent upon mitochondrial membrane potential. After 30 min of incubation, fluorescence was detected with a Perkin Elmer EnSight Multimode Plate Reader (Perkin Elmer, Boston, MA, USA), setting the excitation at 579 nm and the emission at 599 nm.

### 2.7. Statistical Analysis

Data were analyzed and plotted by using Graphpad Prism (Graphpad Software, La Jolla, CA, USA). Statistical significances were assessed by ANOVA followed by Bonferroni multiple comparison test. All data are reported as mean ± standard error of the mean (SEM) and differences between conditions were considered significant at *p* < 0.05.

## 3. Results

### 3.1. Adenosine Is Necessary for Glutamate Cytotoxic Effect in PC12 Cells

**Figure 1:**
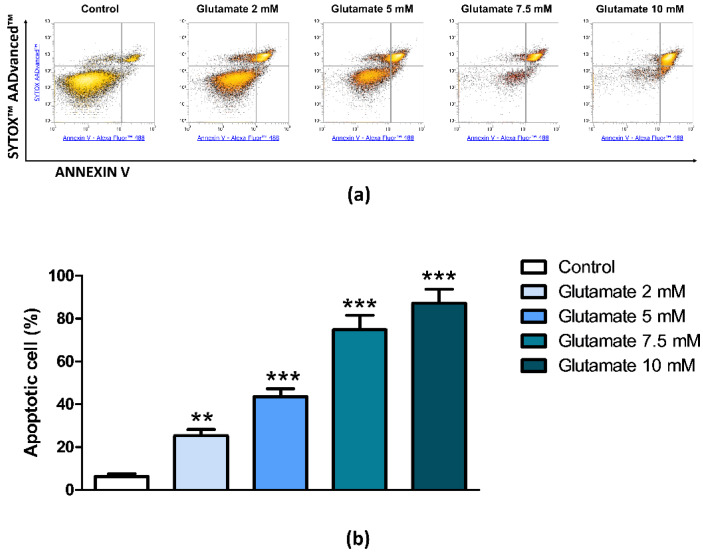
Concentration-dependent cytotoxic effect of glutamate in PC12 cells. (**a**) Representative density plots of flow cytometry analysis of PC12 cells exposed to different concentrations of glutamate for 24 h. Cells were double-stained with Annexin V Alexa Fluor™ 488 Ready Flow Conjugate and SYTOX™ AADvanced™ Dead Cell Stain. Annexin V negative/SYTOX negative cells (bottom left quadrant) represent living cells; Annexin V negative/SYTOX positive cells (top left quadrant) represent necrotic cells; Annexin V positive/SYTOX negative cells (bottom right quadrant) represent early apoptotic cells; Annexin V positive/SYTOX positive cells (top right quadrant) represent late apoptotic cells. (**b**) Histogram showing the percentage of early and late apoptotic PC12 cells. Data are expressed as mean ± SEM of three independent experiments. **, *p* < 0.01 vs. control; ***, *p* < 0.001 vs. control.

Glutamate cytotoxicity is a primary mechanism of neuronal injury following stroke. The role of adenosine and its receptors in an in vitro model of glutamate cytotoxicity in PC12 cells was investigated. The percentage of apoptotic cells was analyzed by flow cytometry measuring the relative number of Annexin V positive PC12 cells subjected to different concentrations of glutamate for 24 h. The tested concentrations (2 mM, 5 mM, 7.5 mM, and 10 mM) determined 25%, 43%, 75%, and 87% of apoptotic cells respectively, indicating a concentration-response effect of glutamate (Figure 1). For the majority of the subsequent experiments, the submaximal concentration of glutamate (7.5 mM) was chosen.

**Figure 2:**
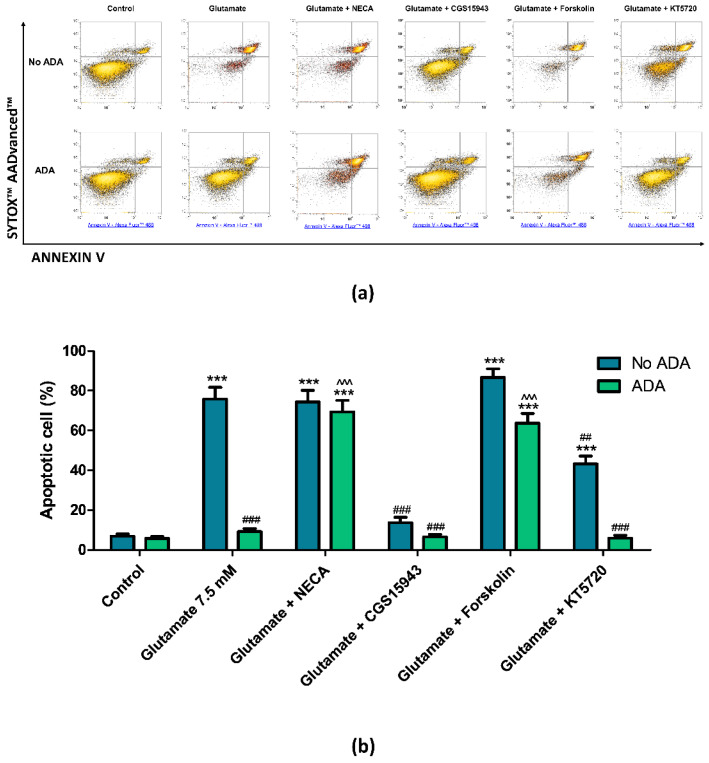
Effect of NECA, CGS15943, Forskolin, and KT5720 on glutamate-induced cytotoxicity in PC12 cells in the absence (No ADA) and the presence of adenosine deaminase (ADA). (**a**) Representative density plots of flow cytometry analysis of PC12 cells exposed to 7.5 mM of glutamate. Cells were pre-treated for 15 min with 10 μM NECA, 10 μM CGS15943, 5 μM Forskolin or 5 μM KT5720 in the absence or the presence of ADA before glutamate exposure. Cells were double-stained with Annexin V Alexa Fluor™ 488 Ready Flow Conjugate and SYTOX™ AADvanced™ Dead Cell Stain. Annexin V negative/SYTOX negative cells (bottom left quadrant) represent living cells; Annexin V negative/SYTOX positive cells (top left quadrant) represent necrotic cells; Annexin V positive/SYTOX negative cells (bottom right quadrant) represent early apoptotic cells; Annexin V positive/SYTOX positive cells (top right quadrant) represent late apoptotic cells. (**b**) Histogram showing the percentage of early and late apoptotic PC12 cells. Data are expressed as mean ± SEM of three independent experiments. ***, *p* < 0.001 vs. control in the absence of ADA; ##, *p* < 0.01 vs. glutamate 7.5 mM in the absence of ADA; ###, *p* < 0.001 vs. glutamate 7.5 mM in the absence of ADA; ^^^, *p* < 0.001 vs. glutamate 7.5 mM in the presence of ADA.

To investigate the involvement of adenosine and its receptors in the cytotoxic effect of glutamate, we first evaluate the contribution of endogenous adenosine using its degrading enzyme adenosine deaminase (ADA). Interestingly, a 15-min pretreatment of PC12 cells with ADA reverted glutamate-induced injury causing a complete abrogation of cell apoptosis (Figure 2). The lack of cytotoxicity in the presence of ADA suggests that endogenous adenosine is a requisite for the glutamate effect. To investigate if the role of adenosine was receptor-mediated, PC12 cells were treated with the non-selective AR agonist NECA at the 10 µM concentration in the absence or in the presence of ADA. NECA mimicked the effect of endogenous adenosine as demonstrated by the increase of the apoptotic rate induced by glutamate in the presence of ADA, reaching a value similar to that obtained by glutamate in the absence of ADA (Figure 2). To further corroborate the receptor-mediated contribution of endogenous adenosine to glutamate cytotoxicity, cells were treated with the non-selective AR antagonist CGS 15943 (10 µM). Blocking the four AR subtypes with CGS 15943 both in the presence or in the absence of ADA resulted in the lack of glutamate-induced apoptosis in a fashion similar to that obtained eliminating endogenous adenosine with ADA (Figure 2). This suggested that the role of adenosine in the glutamate-induce apoptosis is mediated by the activation of ARs. To understand the signaling pathway by which adenosine participated in the glutamate excitotoxic damage, cells were treated with 5 μM forskolin, a specific activator of adenylate cyclase. In the presence of ADA, forskolin re-established the glutamate-induced apoptosis, suggesting that elevated levels of intracellular cAMP are required for the effect of glutamate (Figure 2). This led us to hypothesize that the permissive effect of endogenous adenosine on glutamate cytotoxicity is related to high cAMP levels determined by activation of Gs-coupled AR subtypes. Since protein kinase A (PKA) activity is dependent on cellular levels of cAMP, we tested if PKA inhibition in the presence of endogenous adenosine could interfere with glutamate cytotoxicity. For this purpose, PC12 cells were pre-treated for 15 min with the PKA inhibitor KT5720 before challenging the cells with glutamate in the absence or the presence of ADA. While in the presence of ADA and KT5720 glutamate treatment did not induce apoptosis, in the absence of ADA KT5720 partially but significantly reduced glutamate-induced cytotoxic effect, decreasing the rate of apoptotic cells from 75% to 43% (*p* < 0.01, Figure 2).

### 3.2. The Detrimental Action of Endogenous Adenosine Is Mitigated by Blocking A_2A_ and A_2B_ARs and Further Exacerbated by Blocking A_1_ARs

**Figure 3:**
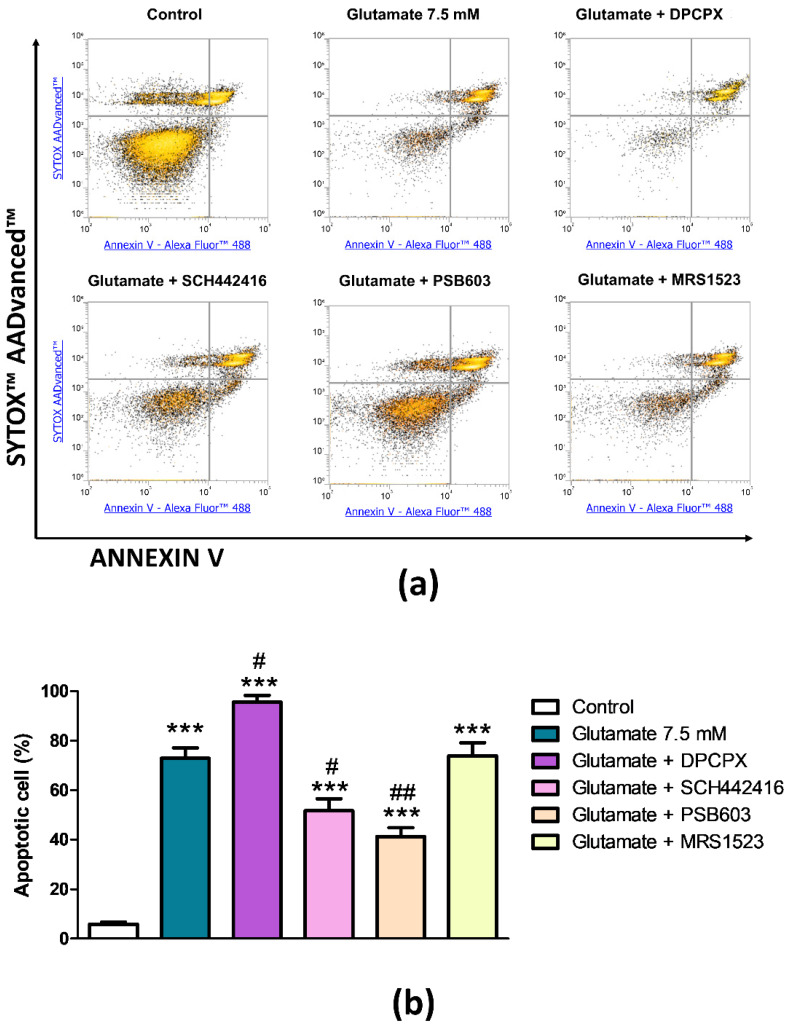
Effect of selective AR antagonists on glutamate-induced cytotoxicity in PC12 cells. (**a**) Representative density plots of flow cytometry analysis of PC12 cells exposed to 7.5 mM of glutamate. Cells were pre-treated for 15 min with 10 μM DPCPX, 10 μM SCH442416, 10 μM PSB603, or 10 μM MRS1523 before glutamate exposure. Cells were double-stained with Annexin V Alexa Fluor™ 488 Ready Flow Conjugate and SYTOX™ AADvanced™ Dead Cell Stain. Annexin V negative/SYTOX negative cells (bottom left quadrant) represent living cells; Annexin V negative/SYTOX positive cells (top left quadrant) represent necrotic cells; Annexin V positive/SYTOX negative cells (bottom right quadrant) represent early apoptotic cells; Annexin V positive/SYTOX positive cells (top right quadrant) represent late apoptotic cells. (**b**) Histogram showing the percentage of early and late apoptotic PC12 cells. Data are expressed as mean ± SEM of three independent experiments. ***, *p* < 0.001 vs. control; #, *p* < 0.05 vs. glutamate 7.5 mM; ##, *p* < 0.01 vs. glutamate 7.5 mM.

To explore the role of the specific AR subtypes in the facilitating action of endogenous adenosine on glutamate cytotoxicity, PC12 cells were pre-treated with selective antagonists before being subjected to glutamate for 24 h. The blockade of A_2A_ARs with SCH442416 significantly reduced glutamate-induced cytotoxicity, decreasing the rate of the apoptotic cells by 31.6% (*p* < 0.05, Figure 3). An even more pronounced effect was obtained using the selective A_2B_ARs antagonist PSB603 that determined a reduction in the percentage of glutamate-induced apoptotic cells by 47.2% (*p* < 0.01, Figure 3). These results confirmed the detrimental action of endogenous adenosine on glutamate cytotoxicity via activation of its Gs-coupled receptor subtypes. We then investigated the contribution of the Gi-coupled receptor. While the blockade of A_3_ARs with MRS1523 did not show any effect on glutamate-induced apoptosis, the presence of the A_1_AR antagonist DPCPX caused a further increase in apoptotic rate exerted by glutamate. As shown in Figure 3, DPCPX exacerbated the cytotoxic effect of glutamate leading the percentage of apoptotic cells to 95.6% (*p* < 0.05). From this data, it is possible to deduce that endogenous adenosine exerts opposite effects on glutamate-induced cytotoxicity, a facilitating action by activating A_2A_ and A_2B_ARs that seems necessary to glutamate to induce apoptosis in PC12 cells, and a protective effect through activation of A_1_AR subtypes as suggested by the exacerbating effect of DPXPC on the apoptotic rate.

### 3.3. Enhancing the Effect of Endogenous Adenosine on A_1_ARs through PAM TRR469 Abrogates the Cytotoxic Action of Glutamate

**Figure 4:**
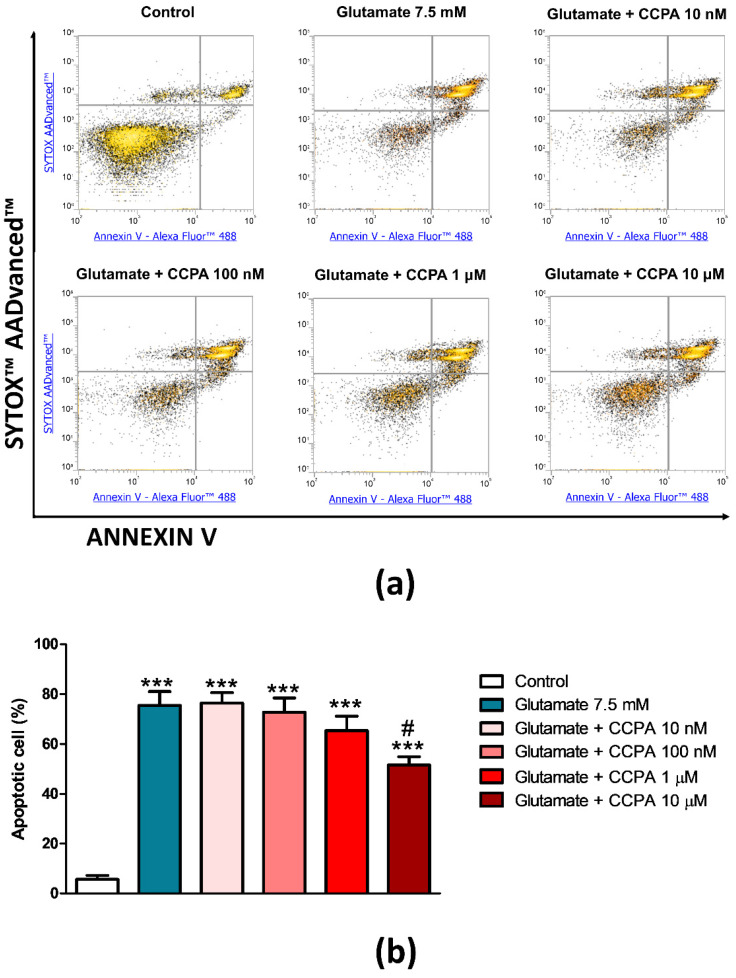
Effect of the A_1_AR agonist CCPA on glutamate-induced cytotoxicity in PC12 cells. (**a**) Representative density plots of flow cytometry analysis of PC12 cells exposed to 7.5 mM of glutamate for 24 h. Cells were pre-treated for 15 min with different concentrations (10 nM–10 μM) CCPA before glutamate exposure. Cells were double-stained with Annexin V Alexa Fluor™ 488 Ready Flow Conjugate and SYTOX™ AADvanced™ Dead Cell Stain. Annexin V negative/SYTOX negative cells (bottom left quadrant) represent living cells; Annexin V negative/SYTOX positive cells (top left quadrant) represent necrotic cells; Annexin V positive/SYTOX negative cells (bottom right quadrant) represent early apoptotic cells; Annexin V positive/SYTOX positive cells (top right quadrant) represent late apoptotic cells. (**b**) Histogram showing the percentage of early and late apoptotic PC12 cells. Data are expressed as mean ± SEM of three independent experiments. ***, *p* < 0.001 vs. control; #, *p* < 0.05 vs. glutamate 7.5 mM.

As a detrimental effect of A_1_ARs blockade on glutamate cytotoxicity has been observed, the potential protective role of A_1_ARs activation by using the selective agonist CCPA at different concentrations (from 10 nM to 10 µM) was investigated. Treatment with CCPA did not show any significant difference in apoptotic rate exerted by 7.5 mM glutamate at all the tested concentrations, with the exception of the 10 µM concentration, which slightly reduced it by 34.2% (*p* < 0.05, Figure 4). This suggests that the A_1_AR agonist alone is not enough to give protection against glutamate injury, probably for the concomitant activation of A_2A_ARs and A_2B_ARs by endogenous adenosine, which exerted a predominant facilitating action on glutamate cytotoxicity.

**Figure 5:**
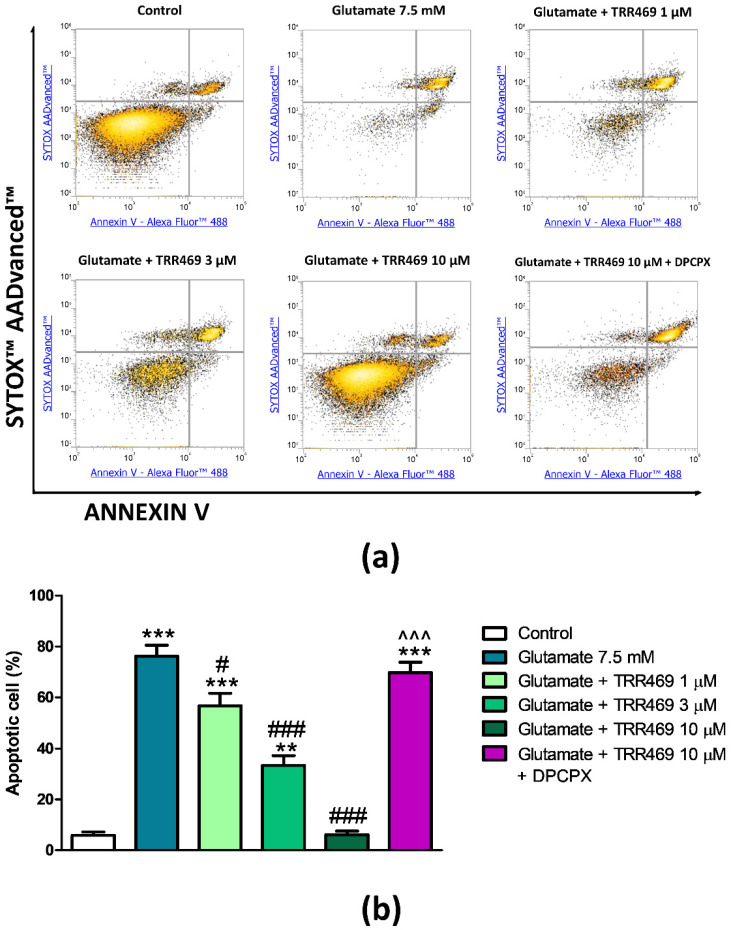
Effect of the A_1_ARs positive allosteric modulator TRR469 on glutamate-induced cytotoxicity in PC12 cells. (**a**) Representative density plots of flow cytometry analysis of PC12 cells exposed to 7.5 mM of glutamate for 24 h. Cells were pre-treated for 15 min with different concentrations (1 μM–10 μM) TRR469 before glutamate exposure. To test the effect of A_1_AR blockade, 10 μM DPCPX was added to the cells 15 min before TRR469. Cells were double-stained with Annexin V Alexa Fluor™ 488 Ready Flow Conjugate and SYTOX™ AADvanced™ Dead Cell Stain. Annexin V negative/SYTOX negative cells (bottom left quadrant) represent living cells; Annexin V negative/SYTOX positive cells (top left quadrant) represent necrotic cells; Annexin V positive/SYTOX negative cells (bottom right quadrant) represent early apoptotic cells; Annexin V positive/SYTOX positive cells (top right quadrant) represent late apoptotic cells. (**b**) Histogram showing the percentage of early and late apoptotic PC12 cells. Data are expressed as mean ± SEM of three independent experiments. **, *p* < 0.01 vs. control; ***, *p* < 0.001 vs. control; #, *p* < 0.05 vs. glutamate 7.5 mM; ###, *p* < 0.001 vs. glutamate 7.5 mM; ^^^, *p* < 0.001 vs. glutamate 7.5 mM + TRR469 10 μM.

Positive allosteric modulation represents an alternative strategy to exploit the protective effect of A_1_AR activation. To verify this hypothesis, PC12 cells were pre-treated with the A1AR PAM TRR469 before the incubation in the presence of 7.5 mM glutamate. We have previously demonstrated that TRR469 increases the affinity and potency of A_1_AR agonists [29]. To enhance the protective effect of endogenous adenosine acting on A_1_ARs, cells were subjected to glutamate insult in the absence or the presence of different concentrations of the A_1_AR positive allosteric modulator TRR 469. The presence of TRR 469 determined a significant and concentration-dependent reduction of glutamate-induced apoptosis, reaching a complete inhibition at the 10 μM concentration (Figure 5). The protective effect of TRR469 was abrogated by the selective A_1_ARs antagonist DPCPX 10 μM, confirming the involvement of this receptor subtype.

### 3.4. TRR469 Prevented Glutamate-Induced Caspase 3/7 Activation

**Figure 6:**
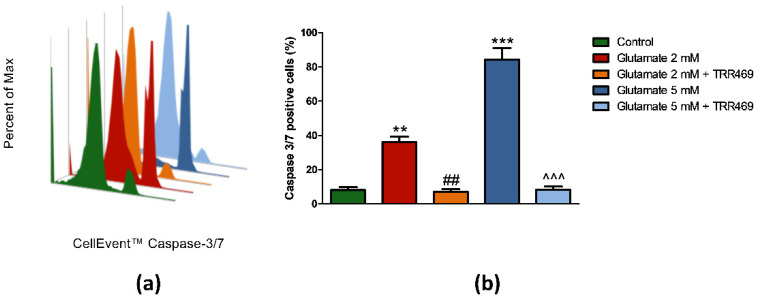
Effect of the A_1_ARs positive allosteric modulator TRR469 on glutamate-induced caspase 3/7 activation in PC12 cells. (**a**) Representative histogram plot overlay of caspase 3/7 fluorescence intensity in PC12 cells exposed to 2 mM and 5 mM of glutamate for 24 h in the absence or the presence of 10 μM TRR469. TRR469 was added 15 min before glutamate exposure. Cells were stained with 1 µM of CellEvent™ Caspase-3/7 Green Detection Reagent 30 min before flow cytometry analysis (**b**) Histogram showing the percentage of caspase 3/7 positive PC12 cells. Data are expressed as mean ± SEM of three independent experiments. **, *p* < 0.01 vs. control; ***, *p* < 0.001 vs. control; ##, *p* < 0.01 vs. glutamate 2 mM; ^^^, *p* < 0.001 vs. glutamate 5 mM.

To better investigate the mechanism by which TRR469 protected PC12 cells from glutamate cytotoxicity, the activation of caspase 3/7, key effectors of the apoptotic pathway, was measured. Glutamate induced an increase of caspase 3/7 positive cells to 35.9% (*p* < 0.01) and 84.3% (*p* < 0.001) from 8.1% in control conditions at the 2 mM and 5 mM concentration, respectively (Figure 6). Exposing the cells to the same concentrations of glutamate in the presence of the positive allosteric modulator TRR469 resulted in significant and complete inhibition of glutamate-induced caspase 3/7 activation (Figure 6).

### 3.5. TRR469 Prevented ROS Production and Mitochondrial Membrane Potential Reduction Induced by Glutamate

**Figure 7:**
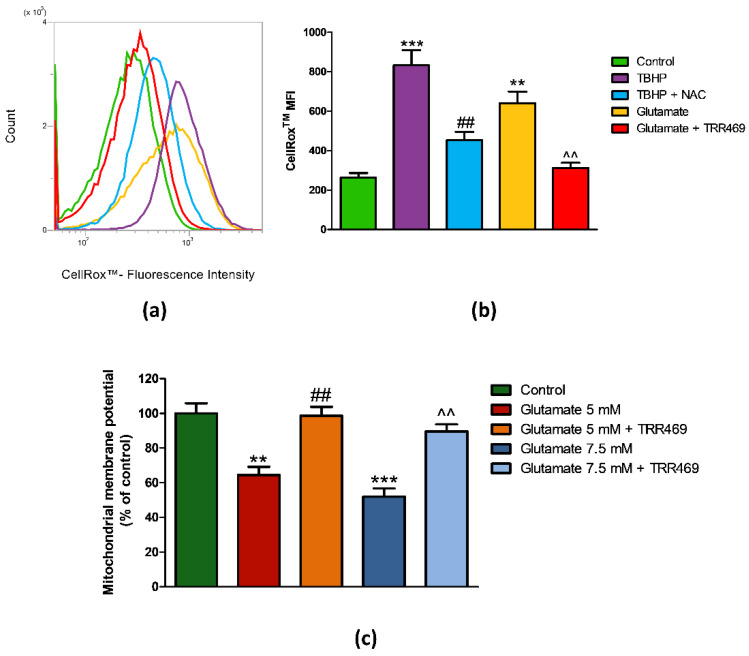
Effect of the A_1_ARs positive allosteric modulator TRR469 on glutamate-induced ROS production and mitochondrial membrane potential loss in PC12 cells. (**a**) Representative histogram plot overlay of CellROX^TM^ fluorescence intensity in PC12 cells exposed to 7.5 mM glutamate for 6 h in the absence or the presence of 10 μM TRR469. TRR469 was added 15 min before glutamate exposure. As a positive control, PC12 cells were treated with 200 μM of the ROS inducer tert-Butyl hydroperoxide (TBHP) for one hour. For the negative control, cells were pre-treated with 2 mM *N*-acetylcysteine (NAC) one hour before TBHP. Cells were stained with 1 µM of CellRox™ Reagent 45 min before flow cytometry analysis. (**b**) Histogram showing the CellRox™ median fluorescence intensity (MFI) in PC12 cells. Data are expressed as mean ± SEM of three independent experiments. **, *p* < 0.01 vs. control; ***, *p* < 0.001 vs. control; ##, *p* < 0.01 vs. TBHP; ^^, *p* < 0.01 vs. glutamate 7.5 mM. (**c**) Mitochondrial membrane potential in PC12 cells reported as MitoTracker™ Red CMXRos fluorescence intensity relative to control (set at 100%). Cells were exposed to 5 mM or 7.5 mM glutamate for 24 h before the incubation for 30 min with MitoTracker™ Red CMXRos. TRR469 was added 15 min before glutamate exposure. Data are expressed as mean ± SEM of three independent experiments. **, *p* < 0.01 vs. control; ***, *p* < 0.001 vs. control; ##, *p* < 0.01 vs. glutamate 5 mM; ^^, *p* < 0.01 vs. glutamate 7.5 mM.

Given that reactive oxygen species (ROS) production is recognized as an early step in glutamate-induced cytotoxicity, we measured ROS level in PC12 cells following 6 h of incubation with 7.5 mM glutamate in the absence and the presence of TRR469. As a positive control for ROS production, 200 μM of the ROS inducer tert-Butyl hydroperoxide (TBHP) was used in the absence and the presence of a 2 mM concentration of the antioxidant *N*-acetylcysteine (NAC). As expected, TBHP significantly increased ROS production, an effect abolished by pre-treating the cells with NAC (Figure 7a,b). ROS levels induced by glutamate reached 66.4% of that produced by TBHP. Interestingly, the presence of TRR469 completely abrogated glutamate-induced ROS production (Figure 7a,b).

Mitochondrial dysfunction is a typical feature of glutamate cytotoxicity. In PC12 cells, glutamate-induced loss of mitochondrial membrane potential by 35.6% (*p* < 0.01) and 48% (*p* < 0.001) in comparison to control conditions, at 5 mM and 7.5 mM concentrations, respectively (Figure 7c). When cells were pre-treated with the A_1_AR positive allosteric modulator TRR469 before glutamate, mitochondrial membrane potential was completely restored to the control condition (Figure 7c).

## 4. Discussion

Glutamate cytotoxicity is a pathological process linked to many neurological diseases and psychiatric disorders [33]. The overactivation of glutamate receptors generates high levels of intracellular Ca^2+^, followed by the generation of ROS and disruption of cell energy production. The resulting depletion of ATP induces a collapse of transmembrane electrochemical gradients, loss of nerve cell function, cell damage, and death primarily by caspase 3/7-mediated apoptosis [34]. Adenosine is an endogenous anti-inflammatory and retaliatory metabolite modulating neurotoxicity both acting on neurons and immune cells expressing ARs G-protein coupled receptor subtypes [35,36]. In the present study, the modulator role of adenosine on glutamate cytotoxicity in PC12 cells has been investigated. PC12 is a cell line derived from rat adrenal medulla pheochromocytoma with the capability to synthesize dopamine and glutamate and has been extensively used as a tool for studying the function of neurons and neurotoxicity [32]. We have previously performed saturation binding experiments on PC12 cells, evaluating the density of adenosine receptors. All four adenosine receptor subtypes were expressed in PC12 cells, with a greater density found for the A_2A_AR subtype in comparison with the other AR subtypes [37]. The data obtained in this work highlight the crucial role of adenosine and its receptors in glutamate cytotoxicity. The lack of endogenous adenosine completely prevented glutamate-induced apoptosis, suggesting that adenosine, through a receptors-mediated mechanism, exerts a facilitating effect on glutamate cytotoxicity. In particular, the presence of endogenous adenosine allowed glutamate-induce cell injury through the activation of its Gs-coupled A_2A_ and A_2B_ARs, as demonstrated by the decrease of apoptotic levels caused by the selective blockade of these receptor subtypes. It is well-known that adenosine, through A_2A_ and A_2B_ARs, stimulates the production of cAMP activating PKA which phosphorylates NMDA channels, thus causing the entrance of Ca^2+^ in the cells leading to neuronal death [38,39]. In the present study, it has been demonstrated that the negative effect of adenosine may be due to the increase in cAMP levels, as confirmed by the restoration, in the absence of endogenous adenosine, of glutamate-induced apoptosis caused by the treatment with forskolin, a direct activator of adenylate cyclase. To corroborate the fundamental role of the cAMP/PKA signaling pathway, when PC12 cells were subjected to glutamate in the presence of the selective PKA inhibitor KT5720, the cytotoxic effect of glutamate was significantly decreased. On the contrary, the presence of the A_1_AR antagonist DPCPX exacerbated glutamate cytotoxicity, suggesting that the stimulation of A_1_ARs by endogenous adenosine could have a protective effect on glutamate-mediated cell injury.

Thus, the antithetical effect on the cAMP/PKA pathway could explain the protective effect of A_1_ARs and the facilitating action of A_2A_ARs and A_2B_ARs on glutamate cytotoxicity. A large body of evidence indicates that adenosine, interacting with A_1_ARs, could act as an endogenous neuroprotective agent since it prevents the damage caused by various pathological conditions, in particular those characterized by an excessive excitatory transmission [9,10,13,16]. In the present study, the treatment with the A_1_ARs agonist CCPA did not fully protect cells from glutamate cytotoxicity, probably due to the concomitant activation of Gs-coupled ARs by endogenous adenosine. Despite their promising therapeutic potential, the use of A_1_AR agonists has been hampered by numerous side effects, poor receptor subtype selectivity, and receptor desensitization [20]. To overcome the problems associated with the utilization of A_1_AR agonists, we developed a series of positive allosteric modulators as a potential alternative for A_1_AR-targeted therapies [22,23,24,25,26,27]. Positive allosteric modulators are an attractive concept in drug targeting because of their potential advantages over conventional agonists. In particular, since positive allosteric modulators can enhance the effect of endogenous agonists, they are expected to have a much lower side effect potential than orthosteric agonists, a low propensity for receptor desensitization and a high receptor subtype selectivity [40,41]. In particular, it is well established that extracellular adenosine levels increase after ischemia or excitotoxic insults in the CNS [8]. Therefore, A_1_AR positive allosteric modulators are expected to have an improved side-effect profile than orthosteric acting agonists because they enhance the effect of elevated adenosine concentration at the site of injury, having little effect on non-pathological sites where adenosine concentration is maintained low. TRR469 is one of the most potent positive allosteric modulators for A_1_ARs so far synthesized. In our previous work, it has been observed the anti-nociceptive effects of TRR469 in models of acute and chronic pain [28] as well as robust anxiolytic-like effects comparable to those of benzodiazepine diazepam [29]. In membranes obtained from the different mouse brain regions, it has been shown that TRR469 was able to greatly increase the affinity of the adenosine analog CCPA. In particular, TRR469 was able to increase CCPA affinity by 17-fold in the hippocampus, 14-fold in the amygdala, and 32 fold in the prefrontal cortex suggesting that the effect the positive allosteric modulation could be retained despite the heterogeneity of the different brain regions [29]. This result is particularly relevant because one of the great advantages of positive allosteric modulators is their ability to increase endogenous agonist affinity, enhancing the activation of the receptor in a more physiological way. The increase of orthosteric ligand affinity by positive allosteric modulation can arise from an increase in ligand association rate and/or a decrease in ligand dissociation rate. At the molecular level, the allosteric two-state model, an extension of the simpler allosteric ternary complex model, describes how the positive allosteric modulator biases the conformational equilibria of GPCR to favor the active state (the agonist high-affinity state) over the inactive one [42]. In this background, we tested the effect of TRR469 in the in vitro model of glutamate cytotoxicity. The results of the present study revealed that TRR469 was able to completely protect PC12 cells from the cytotoxic effect of excessive glutamate concentrations. The effect of TRR469 was concentration-dependent and mediated by the activation of A_1_ARs by endogenous adenosine as demonstrated by the use of the selective antagonist DPCPX that abolished TRR469 cytoprotection. The significantly greater effect of TRR469 respect to the A_1_AR agonist CCPA is most likely due to the enhanced activation of A_1_AR in the presence of the positive allosteric modulator. In particular, while the protective effect of CCPA could be masked by the endogenous adenosine-mediated detrimental activation of A_2A_AR and A_2B_AR, the presence of TRR469, increasing the affinity of adenosine for the A_1_AR subtype, can shift the action of adenosine towards a protective role, overcoming its effect on Gs-coupled subtypes. As the activation of caspases 3 and 7 is a typical hallmark of glutamate-induced cytotoxicity, the reduction of their activation by TRR469 further confirmed the cytoprotective action of the A_1_AR positive allosteric modulator. A large number of works in the literature reports that the neurotoxic effect of glutamate in PC12 cells involves the generation of ROS and mitochondrial dysfunction [43,44,45]. Accordingly, in our experimental conditions, glutamate significantly increased ROS production, an effect that was completely abrogated by the presence of TRR469. Furthermore, the A_1_AR positive allosteric modulator prevented the loss of mitochondrial membrane potential in glutamate-exposed cells. The cytotoxic effect of glutamate in PC12 cells has been primarily linked to oxidative stress rather than direct NMDA receptors activation. This mode of cytotoxicity induced by glutamate in PC12 cells, called oxidative glutamate toxicity, is corroborated by the results obtained in the present work. In particular, the cytotoxic effect in PC12 cells requires elevated concentrations of glutamate, which induces cellular responses typical of the mechanisms involving oxidative stress such as the loss of mitochondrial transmembrane potential and ROS accumulation. The protective effect of TRR469 in PC12 cells suggests the therapeutic potential of A_1_ positive allosteric modulators against glutamate-induced delayed neuronal death that occur independently of receptor activation.

In conclusion, the results of the present work indicate that the A_1_AR positive allosteric modulator TRR 469 as a potentially attractive therapeutic tool in the treatment of neurological disorders characterized by glutamate cytotoxicity. This strategy could allow enhancing the protective effects of endogenous adenosine through A_1_ARs activation possibly avoiding the issue related to the use of A_1_AR agonist.
